# Supplementary material for: RADX condenses single-stranded DNA to antagonize RAD51 loading
Source: Nucleic Acids Res. 2020 Jul 4;48(14):7834–43. doi: 10.1093/nar/gkaa559 (PMC7430644; doi:10.1093/nar/gkaa559)
Supplement: gkaa559_Supplemental_Files [file gkaa559_supplemental_files.zip › Supplements.pdf]

SUPPLEMENTAL INFORMATION

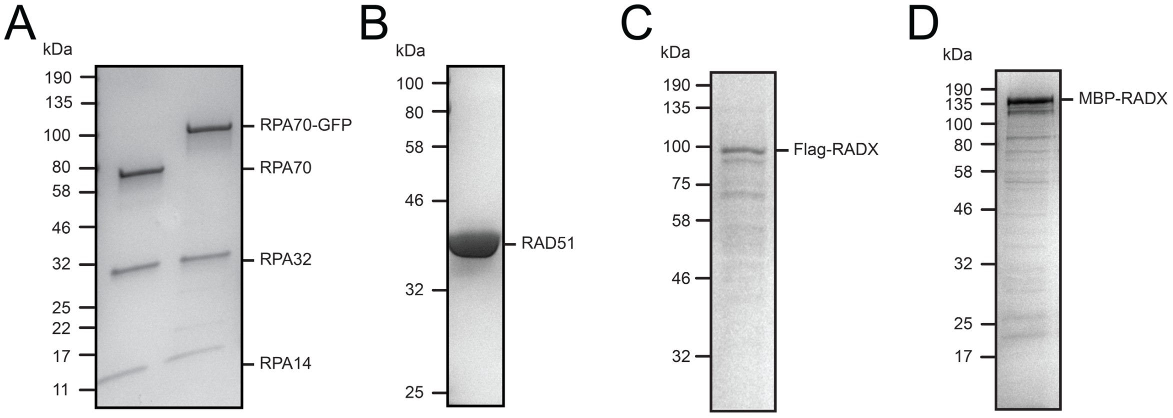

Figure S1. Recombinant proteins used in this study. (A) 4-20% SDS-PAGE gel of human RPA (left) and RPA-GFP (right) purified from *E. coli*. **(B)** 12% SDS-PAGE gel of human RAD51 purified from *E. coli*. 12% SDS-PAGE gels of Flag-RADX **(C)** and His_6-MBP-RADX **(D)** purified from High Five insect cells.

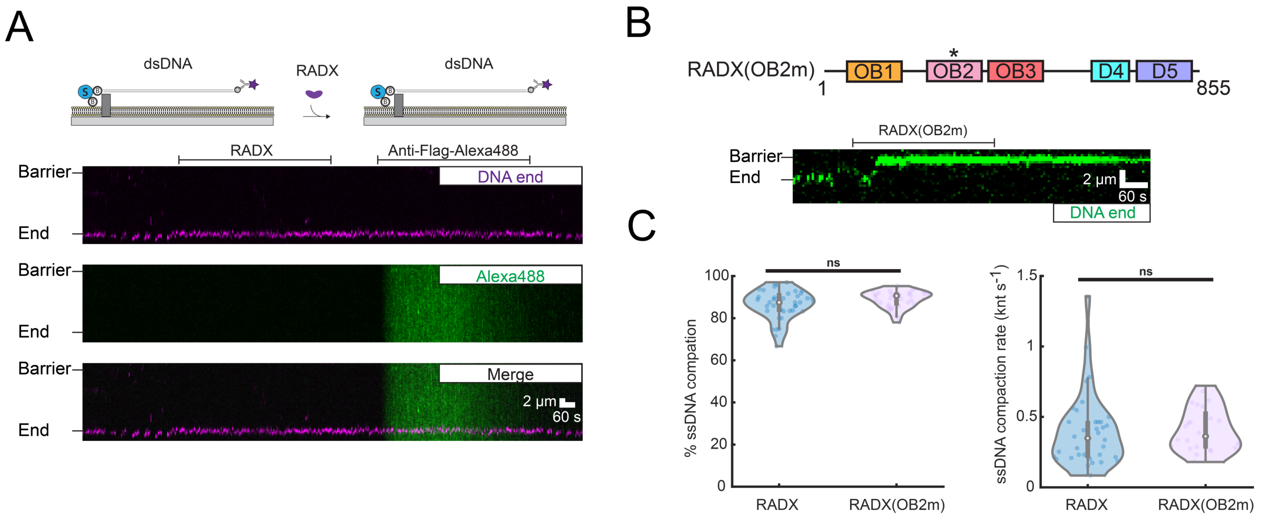

Figure S2. RADX does not bind to dsDNA. RADX(OB2m) compacts ssDNA. (A) A representative kymograph showing RADX does not bind to or compact dsDNA. 2 nM RADX was used in the experiment. **(B)** ssDNA was compacted by 2 nM RADX(OB2m). **(C)** ssDNA compaction percentage and rate in the presence of wtRADX and RADX(OB2m).

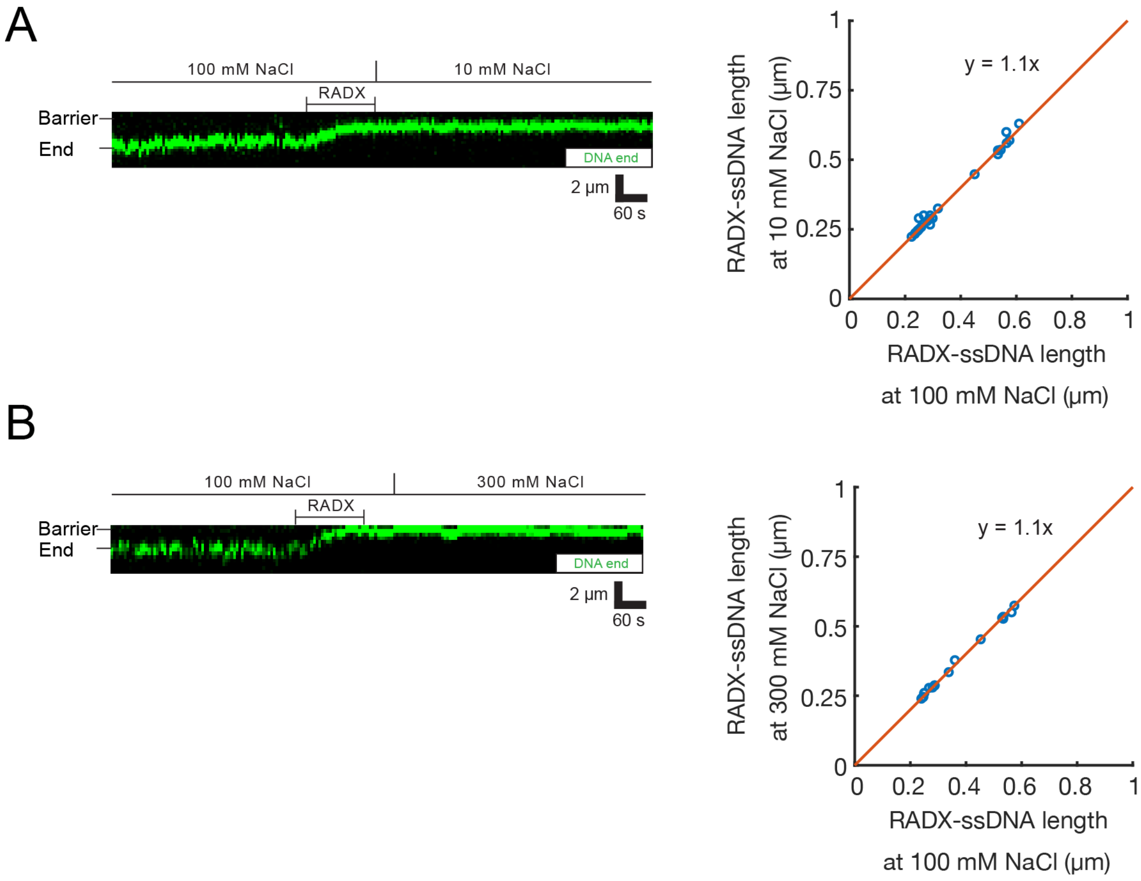

Figure S3. RADX-ssDNA complexes are insensitive to changes in the NaCl concentration. **(A)** A representative kymograph showing NaCl concentration in imaging buffer was switched from 100 mM to 10 mM after ssDNA compacted by 2 nM RADX. Right panel shows the length of RADX-ssDNA before and after salt concentration switch. **(B)** A representative kymograph showing NaCl concentration was switched from 100 mM to 300 mM after ssDNA compacted by 2 nM RADX. Right panel shows the length of RADX-ssDNA before and after salt concentration switch.

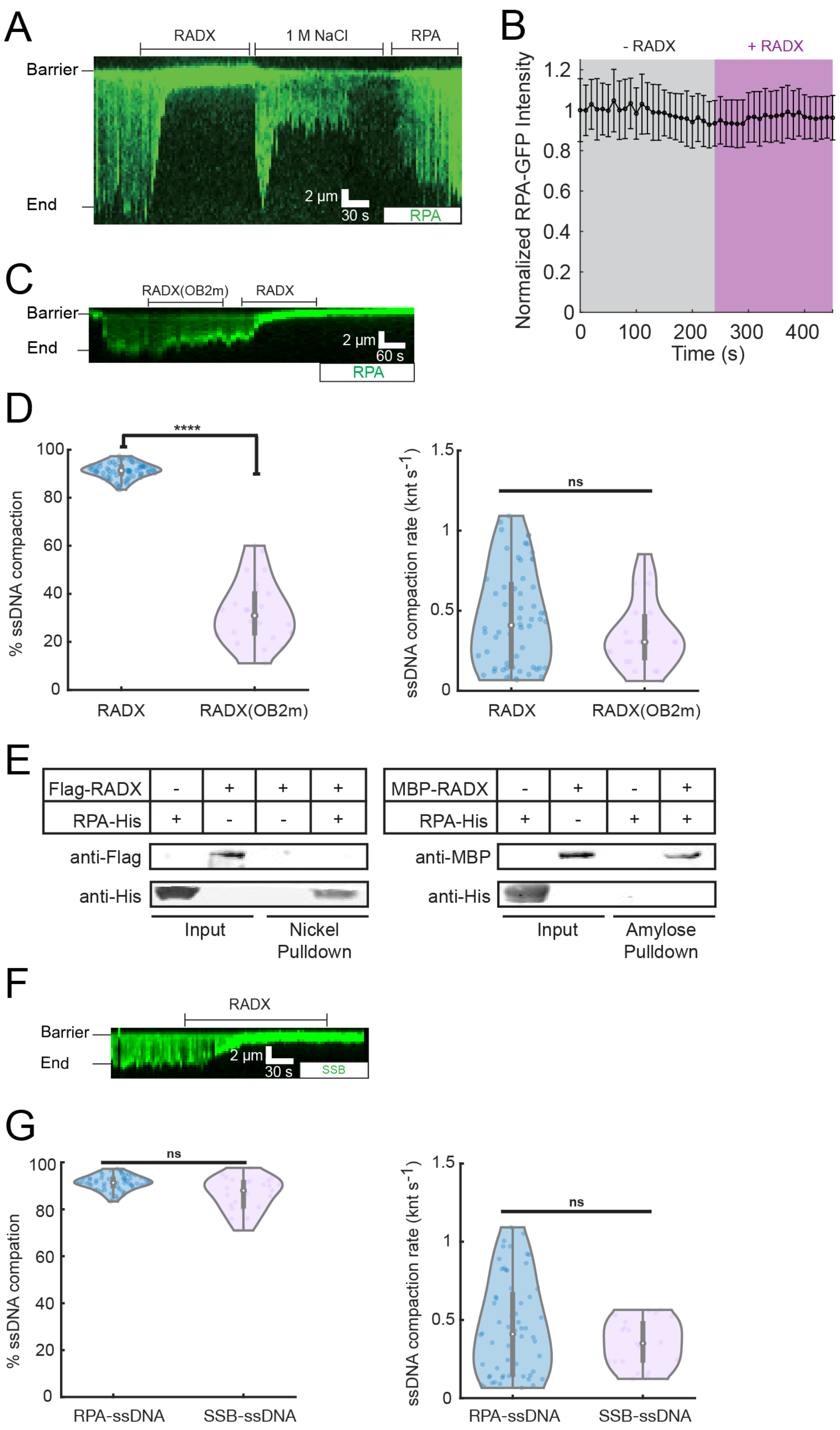
A
RADX
1 M NaCl
RPA
Barrier
End
2 µm
30 s
RPA
B
- RADX
+ RADX
Normalized RPA-GFP Intensity
1.2
1
0.8
0.6
0.4
0.2
0
0
100
200
300
400
Time (s)
C
RADX(OB2m)
RADX
Barrier
End
2 µm
60 s
RPA
D
****
% ssDNA compaction
100
80
60
40
20
0
RADX
RADX(OB2m)
ns
ssDNA compaction rate (knt s-1)
1.5
1
0.5
0
RADX
RADX(OB2m)
E
Flag-RADX
RPA-His
anti-Flag
anti-His
Input
Nickel Pulldown
MBP-RADX
RPA-His
anti-MBP
anti-His
Input
Amylose Pulldown
F
RADX
Barrier
End
2 µm
30 s
SSB
G
ns
% ssDNA compation
100
80
60
40
20
0
RPA-ssDNA
SSB-ssDNA
ns
ssDNA compaction rate (knt s-1)
1.5
1
0.5
0
RPA-ssDNA
SSB-ssDNA

Figure S4. High salt dissociates RADX from ssDNA. RADX(OB2m) has compromised activity to compact RPA-ssDNA. RADX and RPA have no physical interaction. RADX condenses SSB-ssDNA. (A) 1 M NaCl can dissociate RADX from ssDNA and resolve the condensed complexes back to full-length ssDNA molecules. 2 nM RADX was used in the experiment. **(B)** Fluorescent intensity of RPA-GFP on ssDNA before and after RADX injection for experiments in Figure 2A. Magenta shading indicates when RADX enters the flowcell. **(C)** A typical kymograph showing that RADX(OB2m) cannot fully compact RPA-ssDNA substrates. 2 nM RADX(OB2m) was used in the experiment. **(D)** RPA-ssDNA compaction percentage and rate in the presence of wtRADX and RADX(OB2m). **(E)** RPA and RADX do not physically interact, as revealed by reciprocal protein pulldowns. **(F)** 2 nM RADX condenses *E. coli* SSB-coated ssDNA. **(G)** Comparison of SSB-ssDNA and RPA-ssDNA compaction percentage and rate in the presence of RADX.

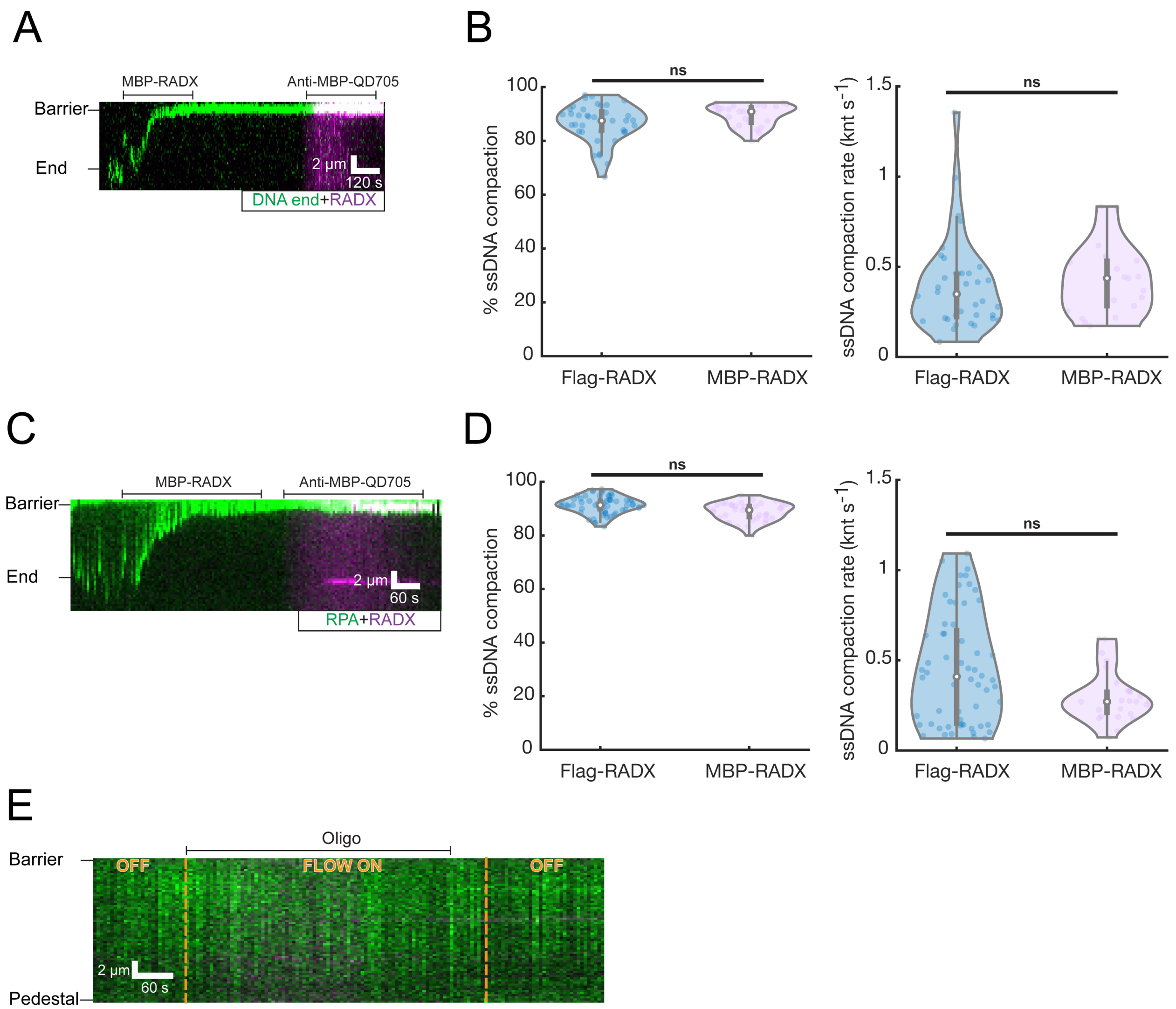

Figure S5. MBP-RADX is functional comparable with Flag-RADX. Non-complementary oligo does not bind to double-tethered RPA-ssDNA. (A) A kymograph showing 2 nM MBP-RADX condenses ssDNA. **(B)** ssDNA compaction percentage and rate in the presence of Flag-RADX and MBP-RADX. **(C)** A kymograph showing 2 nM MBP-RADX condenses RPA-ssDNA. **(D)** RPA-ssDNA compaction percentage and rate in the presence of Flag-RADX and MBP-RADX. **(E)** Non-complementary oligo does not bind to RPA-ssDNA in the absence of RADX.

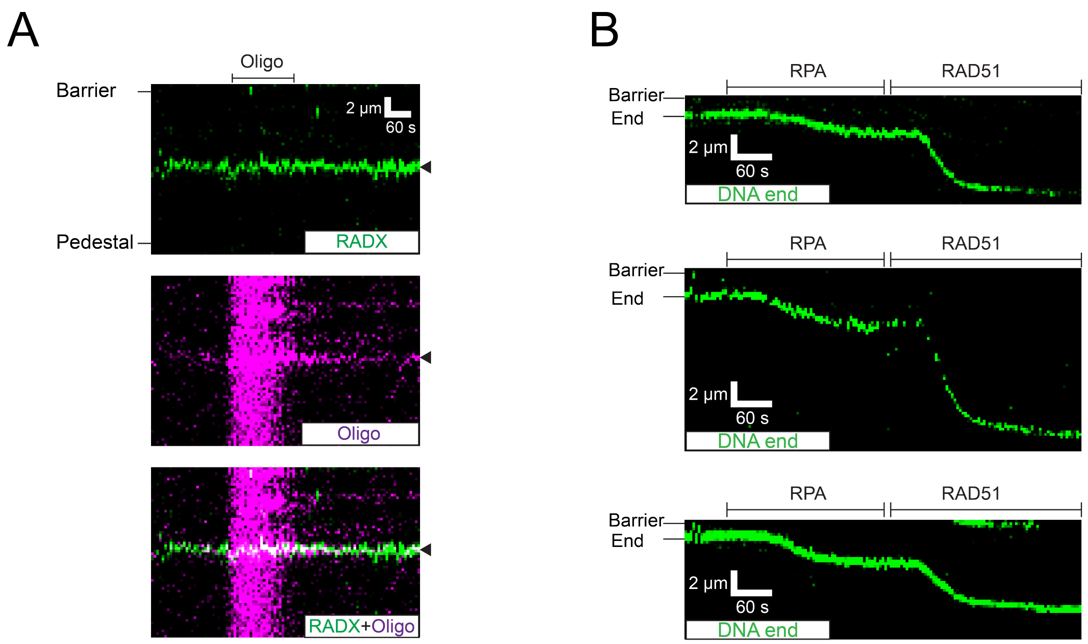

Figure S6. **Representative kymographs that are similar to Figure 3D and Figure 4A bottom. (A)** Additional representative kymograph showing that RADX directly captures non-complementary ssDNA oligonucleotides on RPA-coated ssDNA curtains. Arrows indicate where RADX and oligos bind (see Figure 3D). **(B)** Additional representative kymographs indicating that ssDNA is first bound and extended by RPA and then further extended by RAD51 (see Figure 4A).

SUPPLEMENTAL MOVIES

Movies 1-3. RADX condenses single-stranded DNA. The ssDNA is labeled with an anti-dsDNA antibody (green). RADX (2 nM) is introduced into the flowcell, compacting the ssDNA. DNA-bound RADX is stained with a fluorescent anti-Flag antibody (magenta) after excess protein is flushed out. B: Barrier. E: initial position of the 3'-ssDNA end. Frame rate: 2 s. Scale bar: 2 μm.

Movies 4-6. **RPA-GFP-coated ssDNA is compacted by RADX.** The ssDNA is first saturated with RPA-GFP (green). RADX (2 nM) is introduced into the flowcell, compacting the ssDNA. DNA-bound RADX is stained with a fluorescent anti-Flag antibody (magenta) after excess protein is flushed out. B: Barrier. E: initial position of the 3'-ssDNA end. Frame rate: 2 s. Scale bar: 2 μm.

Table S1. Oligonucleotides used in this study.

Name	Sequence
IF327	GCT GCC GCC CTT GTC ATC
IF328	ACG ATG ACA AGG GCG GCA GCT CCG GAG AGT CTG GGC AAC
IF329	CCT GCA AAG CAC CGG CCT CGT CAG TGG TGG TGG TGG TGG TGA CTA GTA TTT TCA GGA CTG TAA ATC TTG TGA AG
IF330	CAC CAC CAC CAC CAC CAC TGA CGA GGC CGG TGC TTT GCA
IF333	GGA AAA ATC GAA GAA GGT AAA CTG GTA ATC TGG
IF334	CAT ATG TAT ATC TCC TTC TTA AAG TTA AAC AAA ATT ATT TCT AGA GGG G
IF238	5/Biosg/TC TCC TCC TTC T
IF239	/5Phos/AG GAG AAA AAG AAA AAA AGA AAA GAA GG
